# A set of plasmatic microRNA related to innate immune response highly predicts the onset of immune reconstitution inflammatory syndrome in tuberculosis co-infected HIV individuals (ANRS-12358 study)

**DOI:** 10.3389/fimmu.2025.1603338

**Published:** 2025-06-20

**Authors:** Polidy Pean, Ratana Meng, Eliott Benichou, Pichsivannary Srey, Bunnet Dim, Laurence Borand, Olivier Marcy, Didier Laureillard, François-Xavier Blanc, Tineke Cantaert, Yoann Madec, Laurence Weiss, Daniel Scott-Algara

**Affiliations:** ^1^ Unité d’immunologie, Institut Pasteur du Cambodge, Phnom Penh, Cambodia; ^2^ Université Paris-Saclay, Gif-sur-Yvette, France; ^3^ Infectious Diseases Department, Sihanouk Hospital Center of HOPE, Phnom Penh, Cambodia; ^4^ Clinical Research Group, Epidemiology and Public Health Unit, Institut Pasteur du Cambodge, Phom Penh, Cambodia; ^5^ Center for Tuberculosis Research, Division of Infectious Diseases, Johns Hopkins University School of Medicine, Baltimore, MD, United States; ^6^ Research Institute for Sustainable Development (IRD) EMR 271, National Institute for Health and Medical Research (INSERM) UMR 1219, University of Bordeaux, Bordeaux, France; ^7^ Infectious and Tropical Diseases Department, University Hospital, Nimes, France; ^8^ Nantes Université, CHU Nantes, Service de Pneumologie, l’institut du thorax, Nantes, France; ^9^ Epidemiology of Emerging Diseases, Institut Pasteur, Université de Paris, Paris, France; ^10^ Université Paris Cité, Immunology, Paris, France; ^11^ International Affairs Departement, Institut Pasteur, Paris, France

**Keywords:** microRNA, exosomes, HIV, tuberculosis, IRIS, biomarkers

## Abstract

**Background:**

After initiation of combination antiretroviral treatment (cART), HIV-1/tuberculosis coinfected patients are at high risk of developing tuberculosis-associated immune reconstitution inflammatory syndrome (TB-IRIS). MicroRNAs, small molecules of approximately 22 nucleotides, which regulate post-transcriptional gene expression and their profile has been proposed as a biomarker for many diseases. We tested whether the microRNA profile could be a predictive biomarker for TB-IRIS.

**Methods:**

Twenty-six selected microRNAs involved in the regulation of the innate immune response were investigated. Free plasmatic and microRNA-derived exosomes were measured by flow cytometry. The plasma from 74 HIV-1+TB+ individuals (35 IRIS and 39 non-IRIS) at the time of the diagnosis and before any treatment (baseline) of CAMELIA trial (ANRS1295-CIPRA KH001-DAIDS-ES ID10425); 15 HIV+TB− and 23 HIV−TB+, both naïve of any treatment; and 20 HIV−TB− individuals as controls were analysed.

**Results:**

At baseline, both IRIS and non-IRIS HIV+/TB+ individuals had similar demographic and clinical characteristics, including sex, age, body mass index, very low CD4+ cell counts (27 cells/mm^3^), and plasma HIV RNA load levels (5.76 log copies/ml). Twenty out of 26 plasmatic-microRNAs tested were no different between IRIS and controls. Twelve of the 26 tested microRNAs showed statistically significant differences between IRIS and non-IRIS patients (p-values ranging from p <0.05 to p <0.0001). Among these, five could discriminate between IRIS and non-IRIS individuals using ROC curve analysis (AUC scores ranging from 0.74 to 0.92). The combination of two (hsa-mir-29c-3p and hsa-mir-146a-5p) or three microRNAs (hsa-mir-29c-3p, hsa-mir-29a-3p, and hsa-mir-146a-5p) identified IRIS with 100% sensitivity and high specificity (95% and 97%, respectively).

**Conclusion:**

The combination of at least two or three plasmatic microRNAs known to regulate inflammation and/or cytokine responses could be used as biomarkers to discriminate IRIS from non-IRIS in HIV-TB co-infected individuals at the time of diagnosis and prior to any treatment.

## Introduction

MicroRNAs are endogenous non-coding RNA, small single-stranded RNAs typically 22 nucleotides in length. MicroRNAs are powerful regulators of post-transcriptional gene expression (1). They could be a potential biomarker in many diseases, including infectious diseases (2, 3). microRNAs were initially identified in *Caenorhabditis elegans* and later recognized for playing pivotal roles in a vast range of cellular functions (4–6). It has also been shown that miRNAs, by their capacity to regulate gene expression, play a crucial role not only in the host’s defense against viruses but also in facilitating the establishment of viral infections (7).

During human immunodeficiency virus type 1 (HIV-1) infection, host microRNA profiles (cellular or circulating) are altered either to control the virus or as a mechanism for the virus to facilitate viral replication, and infection or to maintain latency (7–9). Additionally, microRNA-like elements coded by pathogens including HIV-1, have been described (8, 10). Several studies have reported that host microRNAs target either directly or indirectly HIV genes to control viral replication and disease progression (9, 11). Interactions between cellular microRNAs and HIV-1 have been reported such as the modulation of the expression of cellular proteins essential to the viral cycle known as HIV dependency factors (HDF) (11), or as inhibition of the expression of HIV Nef protein (12). microRNA profiles can also characterize clinical stage of HIV infection. In a group of 8 Elite Controllers compared to 8 viremic progressors, Egaña-Gorroño et al. found a pattern consisting of 23 microRNAs: 4 microRNAs were overexpressed (hsa-miR-221, -27a, -27b and -29b), while the other 19 were down-regulated (13). One study describing the profile of microRNA in acute and chronic HIV infection showed a strong activation of innate immune response (14).

In HIV-TB coinfection, the Immune Reconstitution Inflammatory Syndrome (TB-IRIS) is an pathological inflammatory syndrome that occurs in a subset of severely HIV+ immunosuppressed patients. It typically occurs after the initiation of combined antiretroviral treatment (cART), which leads to the restoration of immune responses (15, 16).

On the other hand, several plasmatic microRNAs profiles in individuals infected with *Mycobacterium tuberculosis* have been characterized, but common biomarkers associated with tuberculosis infection (TB) have not been yet identified. The microRNA expression profiles of PBMCs among patients with active TB, subjects with latent TB infection, and healthy controls have been compared using microarray-based expression profiling followed by real-time quantitative PCR validation (17, 18). These reports showed the potential use of various microRNAs, including hsa-mir-365, hsa-mir-223, hsa-mir-144, hsa-mir-451, hsa-mir-424, miR-155 and miR-155* etc. Target prediction and interaction networks have linked microRNA and mRNA expression data, that target the potential mitogen-activated protein kinase family (MAPK) signalling and other critical immune response pathways, including T cell-receptor, Toll-like receptor and Nod-like receptors signalling pathways (17, 18). Other study showed that miR-29* had potential as a biomarker for active disease (19). Fu et al. studied circulating microRNAs in patients with active pulmonary tuberculosis and found differential expression of certain microRNAs during active pulmonary tuberculosis infection (20).

Thus, circulating microRNAs are promising as sensitive biomarkers for several diseases. microRNAs can be secreted into the extracellular space in microvesicles (21–23), ectosomes (21–23), exosomes (21–23) or microvesicles (such as liposomes) (21–23). In the circulation, microRNA may be associated with high-density lipoproteins (24). Exosomes are small vesicles, 30–150 nm in diameter, surrounded by a lipid membrane bilayer and secreted by most cells in the body (22, 25). They carry several molecules, including microRNA (22, 25). microRNAs are the most concentrated cargo molecules in the exosome (26). Exosomes containing microRNA play a role in the post-transcriptional regulation of gene expression by targeting mRNA and can be taken up in a variety of ways by neighbouring or distant cells. Therefore, to understand the physiopathology of several diseases, it may be important to study the microRNA profile in exosomes. There is also a lack of studies on microRNAs in HIV co-infections such as tuberculosis. In this study, we investigated whether circulating microRNAs could be used as potential biomarkers in diseases associated with HIV-TB co-infection, specifically IRIS. We aim to study the microRNA expression profile in a cohort of patients with HIV infection and tuberculosis and correlate it with their clinical evolution and the occurrence of IRIS.

## Methods

### Individuals and samples

This study is based on CAMELIA clinical trial and CAPRI-NK studies. The CAMELIA clinical trial (ANRS1295-CIPRA KH001-DAIDS-ES ID10425) was a prospective, randomized, multicentre, open-label, 2-arm superiority trial conducted in Cambodia, which demonstrated markedly improved survival when cART was initiated at 2 weeks versus 8 weeks after TB treatment in HIV/TB co-infected patients with CD4 cells < 200 cells/mm^3^ (27). Early cART was also associated with a significantly increased risk of tuberculosis associated IRIS (TB-IRIS). The CAPRI NK study (ANRS12153) is an immunological study related to the CAMELIA clinical trial. We showed that the innate immune response plays a role in TB-IRIS and that Natural Killer cell degranulation capacity could be a predictor of TB-IRIS (28). The frozen plasma samples collected during the CAMELIA and CAPRI NK studies were used in this study after obtaining the approval from National Institute of Health and Medical Research (Inserm) ANRS Emerging infectious diseases (ANRS-MIE, France) as the sponsor of the studies and the National Ethical Committees for Health Research of Cambodia (NECHR). TB and HIV mono-infected individuals were enrolled at the Sihanouk Hospital Center of HOPE in Cambodia. Twenty healthy individuals were recruited at the Institut Pasteur du Cambodge (pre-marital test for HIV). The study was carried out in compliance with the protocol and in accordance with: (i.) Relevant national guidelines for health research (e.g. NECHR guidelines), (ii.) the Declaration of Helsinki approved by the World Health Association on June 1964 amended in Tokyo 1975, Venice 1983, Hong Kong 1989, Somerset West 1996, Edinburgh, Scotland, 2000, Tokyo 2004, (iii.) the recommendations of the Good Clinical Practices (ICH Harmonized Tripartite Guidelines for Good Clinical Practice E6 step 4 - 1996), and (iv.) the ANRS-MIE Ethics charter for research in developing countries (May 2002, amended October 2008). Consent forms were signed by all participants before any collection of data or samples. IRIS diagnosis is defined as indicated in the CAMELIA clinical trial protocol (27). The demographic and clinical variables of the cohort and control groups are shown in **Table 1**.

**Table 1:** Demographic and clinical characteristics of the study participants stratified by HIV and TB status, Including IRIS and Non-IRIS groups.

	HIV+/TB+ (n=69)		Controls		
	IRIS (n= 35)	non IRIS (n= 34)	HIV-TB+ (n=23)	HIV+TB- (n=20)	HIV-TB- (n=20)
Age, yrs					
Median (25-75%IQR)	34 (28-40)	35 (31-44)	38 (31-55)	34 (27-45)	31 (27-35)
Gender					
Male, n (%) Female , n (%)	23 (66) 12 (34)	19 (56) 15 (44)	10 (43) 13 (57)	12 (60) 8 (40)	14 (70) 6 (30)
BMI, kg/m^2^					
Median (25-75%IQR)	16.0 (15.4-18.4)	16.8 (15.6-19.0)	19.1 (17.6-19.7)	19.1(18.1-22.1)	23.7 (22.3-25.4)
CD4, cells/mm^3^					
Median (25-75%IQR)	27 (9-47)	27 (10-68)	NA	91(34-173)	NA
Viral load, log copies/ml					
Median (25-75%IQR)	5.8 (5.4-6.0)	5.5 (5.0-5.8)	–	–	–
Clinical form of TB					
Pulmonary TB, n (%) Disseminate TB , n(%)	20 (57) 15 (43)	27 (79) 7 (21)	23 (100) 0 (0)	–	–

Data are presented as median (interquartile range, IQR) and number (percentage, %). IRIS, immune reconstitution inflammatory syndrome; TB, tuberculosis; HIV, Human Immunodeficiency Virus; BMI, body mass index; CD4, CD4+ T-cell count; NA, not applicable.

### Plasma-derived exosomes purification

Briefly, 1 ml of plasma sample was initially centrifuged at 1500 g for 10 min and then at 10,000 g for 10 minutes to remove cells and large debris. The plasma supernatant was subjected to size-exclusion chromatography and fractionated with qEV original columns 35nm by automatic fraction collector (IZON Science LTD., Cambridge, UK) according to manufacturer’s instructions. Exosome-containing fractions were concentrated using an Amicon Ultra-2 centrifugal filter 10K device (Merck KGaA, Darmstadt, Germany) for subsequent analysis. We verified the percentage of exosome purification by using the MACSPlex human EV capture beads (Miltenyi Biotec B.V. & Co. KG, Germany) following the manufacturer’s instructions. After incubation of exosome captured beads with labelled CD9, CD63 and CD81 monoclonal antibodies, we measured the mean fluorescence intensity (MFI) of each marker by flow cytometry analysis.

### microRNA profiling in plasma and plasma-derived exosomes

Twenty-six microRNAs, known for their putative role in HIV and TB infection, were selected. The selection of microRNAs implicated in innate immune responses was conducted by searching in MEDLINE, Scopus, and Web of Science libraries for publications between Jan 1, 2010, and Dec 31, 2016.

The FirePlex™ miRNA assay was performed in a 96-well filter plate, according to the manufacturer’s instructions (Abcam, London, UK). In brief, plasma or purified exosomes samples were digested with lysis buffer and then incubated with microRNA-specific probes embedded in FirePlex™ hydrogel particles at 37°C for 60 minutes for hybridization. Then, the captured microRNAs were ligated with the universal adaptor sequences present in labelling buffer for subsequent amplification by PCR with labelled primers. The ligated microRNAs were re-hybridized to the probes at 37°C for 30 minutes. Then, the fluorescence tag (e.g. reporter) were added and analysed by a BDFACSCanto II flow cytometer (BD Bioscience, Paris, France). Positive controls were particles bear probes for a miRNA-like target, X-control, which is present in FirePlex Buffer at a concentration of ~1 fmol per 25 μl. This control provides confidence that the assay was successfully implemented in every well. Blank particles bear no probe, providing a baseline level of background fluorescence in every assay well. The following endogenous controls were used: RNU44, RNU48, RNU6B miR-451a, miR-16 and miR-486 (Humans). The samples were analyzed by FirePlex™ Analysis Workbench software (Abcam, London, UK).

### Statistical analysis

Significant differences between the studied group were assessed based on their Bonferroni-corrected adjusted p-value using the *FirePlex™ Analysis Workbench software*. Subsequent statistical analyses were performed using GraphPad Prism 10 software (GraphPad Software, Boston, Massachusetts USA, www.graphpad.com). The non-parametric, Mann-Whitney U test and Wilcoxon matched-pairs signed rank test were employed. A p-value of ≤ 0.05 was considered statistically significant.

To evaluate the discriminative power of each microRNA marker between the IRIS and non-IRIS groups, we constructed Receiver Operating Characteristic (ROC) curves displaying area under the curve (AUC) with 95% confidence interval (CI) using GraphPad Prism version 10 software (GraphPad Software, CA, USA). Additionally, we conducted a combinatorial analysis of various plasmatic microRNA marker levels to determine the optimal combination markers for differentiating between the IRIS and non-IRIS groups. This analysis utilized CombiROC, following the guidelines provided by the authors (29). The CombiROC method is an interactive, free web-based statistical tool available at http://combiroc.eu/. We systematically explored all possible microRNA marker combinations between the IRIS and non-IRIS groups. Furthermore, we assessed the performance of selected microRNA ROC curves using an interactive free web tool (29). To compare the level of plasma microRNA at the IRIS onset, each IRIS participant was paired with one non-IRIS control whose plasma microRNA results were available within 14 days of the case values. The Wilcoxon matched-pairs signed rank test was employed for the comparison.

## Results

### IRIS individuals exhibit a distinct microRNA profile in plasma compared to non-IRIS at baseline before any treatment

**Figure 1:**
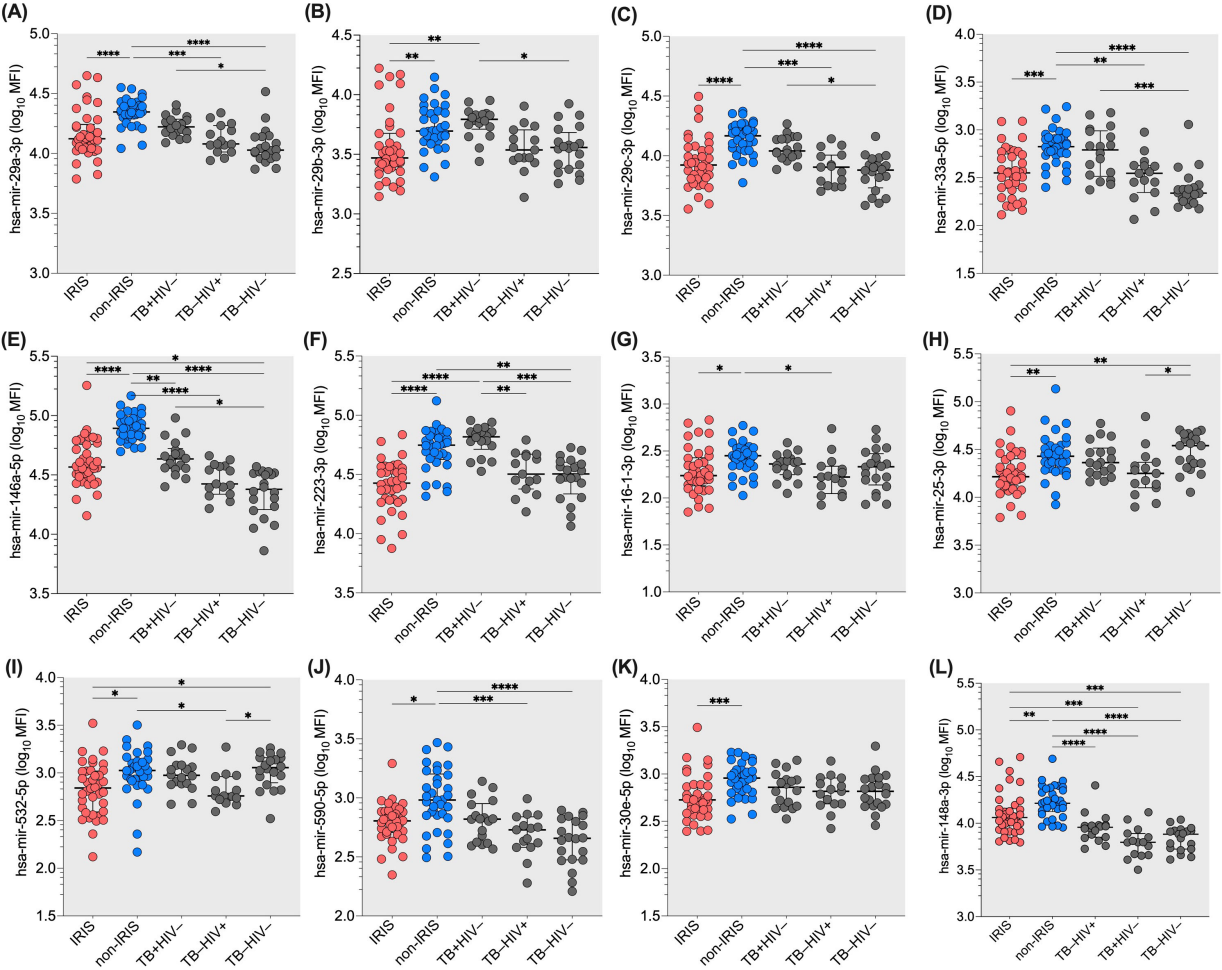
Differential expression of twelve plasma microRNAs in IRIS and non-IRIS patients at baseline and controls. The expression level of the tested plasma microRNAs is indicated on the Y-axis. The X-axis represents the study groups. Lower plasma levels were found in IRIS individuals compared to non-IRIS individuals. Non-IRIS individuals have higher plasma levels for the twelve microRNAs : **(A)** hsa-mir-29a-3p; **(B)** hsa-mir-29b-3p; **(C)** hsa-mir-29c-3p; **(D)** hsa-mir-33a-5p; **(E)** has-mir-146a-5p; **(F)** hsa-mir-223-3p; **(G)** hsa-mir-16-1-3p; **(H)** hsa-mir-25-3p; **(I)** hsa-mir-532-5p; **(J)** has-mir-590-5p; **(K)** hsa-mir-30e-5p; and **(L)** has-mir-148a-3p. In contrast, IRIS patients have similar levels compared to TB and HIV controls. Kruskal-Wallis test was used for analysis. Results are presented as median (25%-75% IQR) log_10_MFI. Statistically significant difference is indicated: * p<0.05; ** p=0.01; *** p=0.001; and **** p<0.0001.

To compare the microRNA profile in plasma of IRIS (n=35) and non-IRIS (n=39) participants, we selected a specific panel of 26 microRNAs (**Supplementary Table S1**). These microRNAs are known for their regulation of innate immune response and inflammation as well as their targeting of genes related to innate immunity, supported by previous results from our group and others (30). All the individuals included in the study are naïve of treatment for both HIV and/or TB infections. The significant differences in microRNA profiles generated by the FirePlex analysis are shown in **Figure 1**. **Figure 1A-L** represents the microRNAs that exhibit statistical differences between IRIS and non-IRIS participants. Notably, we identified 12 microRNAs, including hsa-mir-29a-3p; hsa-mir-29b-3p; hsa-mir-29c-3p; hsa-mir-33a-5p; hsa-mir-146a-5p; hsa-mir-223-3p; hsa-mir-16-1-3p; hsa-mir-25-3p; hsa-mir-532-5p; hsa-mir-590-5p; hsa-mir-30e-5p and hsa-mir-148a-3p that were found statistically different between the two groups (the *p-value* range from p=0.02 to p<0.0001). Of note, non-IRIS participants exhibited similar or higher levels for these 12 microRNAs compared to TB mono-infected individuals and higher levels for most of them when compared to HIV mono-infected or control individuals (**Figures 1A-L**). For the other 14 plasma microRNAs tested simultaneously, we found no difference between IRIS and non-IRIS participants (**Supplementary Figure S1**).

**Figure 2:**
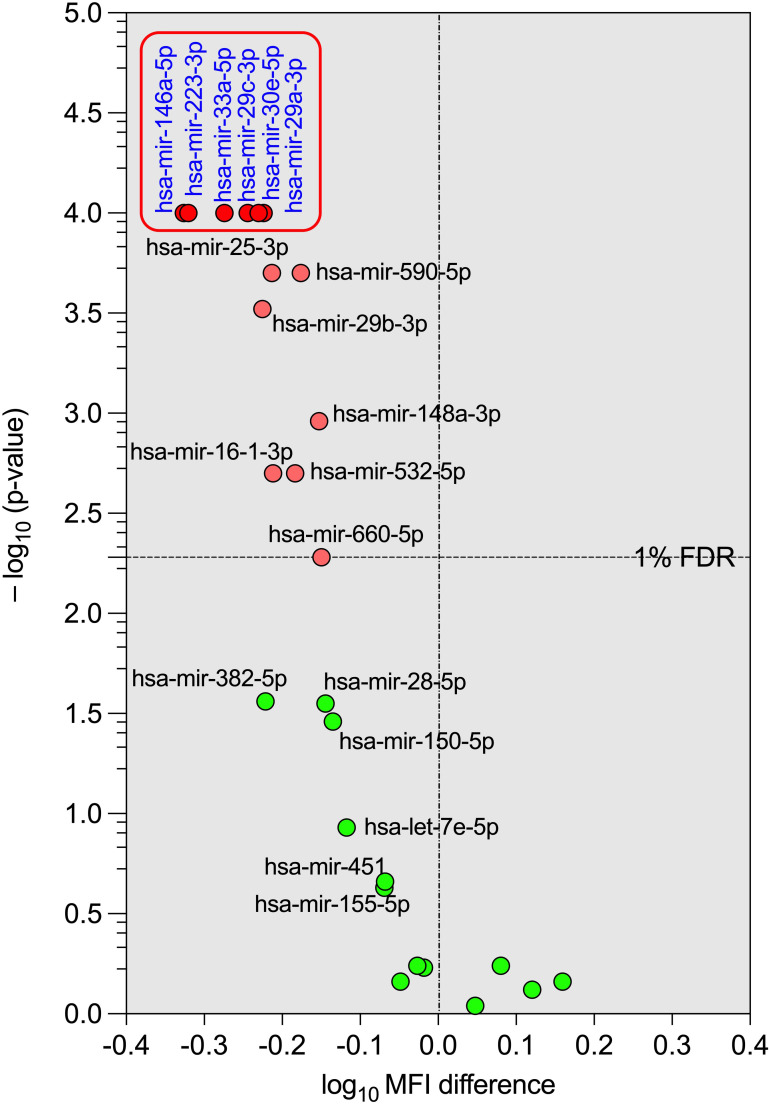
Volcano plot of the differential expression of the tested plasma microRNAs between IRIS and non-IRIS patients at baseline. The circular dots represent the differences in the median value of each plasma microRNA level and its p-value in transformed -log_10_ between the IRIS and non-IRIS groups. The name of each microRNA is shown on the graph. The x-axis shows the log_10_ MFI difference in plasma microRNA levels between the IRIS and non-IRIS groups, while the y-axis shows the distribution of p-values (transformed to -log_10_) obtained from the statistical comparison between the IRIS and non-IRIS groups for each plasma microRNA. A control false discovery rate (FDR) of 1%, represented by a horizontal dotted line, was established to determine the small p-values of the corresponding comparison using the Benjamini method for stacked p-value analysis (GraphPad Prism v10). Significantly different levels are represented by red dots. Among these, six plasma microRNAs with p-values less than 0.0001, shown in the square box, were selected for further analysis.

To visualise the results of the differential expression analysis of the microRNA profiles in IRIS and non-IRIS participants, we constructed a volcano plot (negative logarithm of the p-value on the y-axis and logarithm of the difference of microRNA expression level between IRIS and non-IRIS on the x-axis) with a cut-off at 1% False Discovery Rate (FDR). As shown in **Figure 2**, the most statistically significant microRNAs are at the top and are downregulated (upper-left quadrant of the volcano plot) in IRIS participants (six microRNAs are indicated). These six microRNAs are among the 12 already identified in our first analysis (**Figure 1**). This analysis confirms the strong difference in plasma microRNAs levels in IRIS participants compared to non-IRIS participants. The representative heatmap of the plasma microRNA levels between IRIS and non-IRIS individuals is shown in **Supplementary Figure S4**.

The alteration of microRNA derived plasma exosome in HIV co-infection with tuberculosis have been reported (32, 33) and could be a diagnostic marker of tuberculosis (32). We next assessed the expression of exosomes markers CD9; CD63 and CD81 and microRNA content in the purified exosomes from plasma samples collected during the CAPRI-NK study. The exosome markers of HIV and TB mono-infected individuals have higher significant MFI values compared to HIV-TB co-infected individuals (IRIS or non-IRIS) and healthy donors (**Supplementary Figure S2A**). For CD9 and CD81, HIV-TB (IRIS or non-IRIS) infected individuals have similar MFI to healthy donors (**Supplementary Figure S2A**). The CD63 marker MFI was high in non-IRIS participants compared to healthy controls (p ≤ 0.01), but there is no difference between IRIS et non-IRIS participants for the three markers. Unlike plasmatic microRNA, exosomal microRNA profile showed no significant differences between IRIS and non-IRIS participants (**Supplementary Figure S2B**).

### At the time of IRIS, six plasmatic microRNA are different between IRIS and non-IRIS matched participants

**Figure 3:**
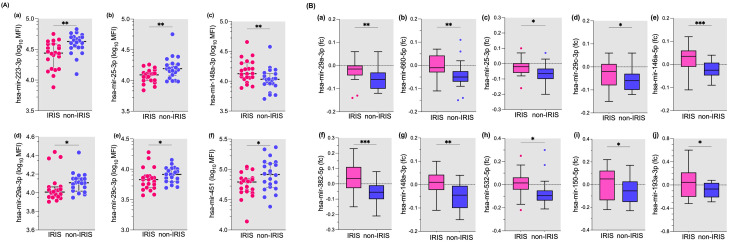
Plasma levels of microRNAs at IRIS onset and fold change from baseline to IRIS onset in IRIS versus non-IRIS patients. **(A)** Plasma levels of six of the 26 microRNAs tested are shown. These microRNAs were significantly different between IRIS and non-IRIS subjects. **(B)** The fold change of ten microRNAs that were significantly different between IRIS and non-IRIS individuals are shown. The Wilcoxon matched-pair test was used for analysis and results are presented as median (25%-75% IQR). Statistically significant differences between study groups are indicated as follows: *p<0.05; **p=0.01; ***p=0.001.

In the next set of experiments, we compared the plasmatic levels of 26 microRNAs in 22 IRIS patients and 22 non-IRIS matched individuals at the time of IRIS occurrence (28). Six out of 26 microRNAs were significantly different between IRIS compared with non-IRIS at the time of IRIS onset (**Figure 3A**). Five microRNAs were lower in IRIS patients compared to non-IRIS controls (**Figure 3A**), and has-mir-148a-5p was significantly higher in IRIS patients (p = 0.01) (**Figure 3A**).

For the remaining 20 microRNAs tested, no significant differences were found between IRIS and non-IRIS subjects (**Supplementary Figure S3A**). When we analysed the fold change from baseline to the onset of IRIS, the change in plasma levels of 10 microRNAs showed a significant difference between the IRIS and non-IRIS groups (**Figure 3B**). Compared to non-IRIS subjects, IRIS subjects show relatively stable plasma levels between baseline and IRIS onset, whereas non-IRIS patients have decreased plasma levels for all ten microRNAs (**Figure 3B**). The change in plasma levels of the remaining 16 microRNAs was not significantly different between the two groups (**Supplementary Figure S3B**).

When we compared the median fold change values of eight microRNAs between IRIS and non-IRIS individuals, we observed a trend towards the lowest values in the non-IRIS individuals, although the differences were not significant (**Supplementary Figure S3B**).

### A set of two or three plasmatic miRNAs highly predicts onset of IRIS in HIV – TB coinfected individuals

**Figure 4:**
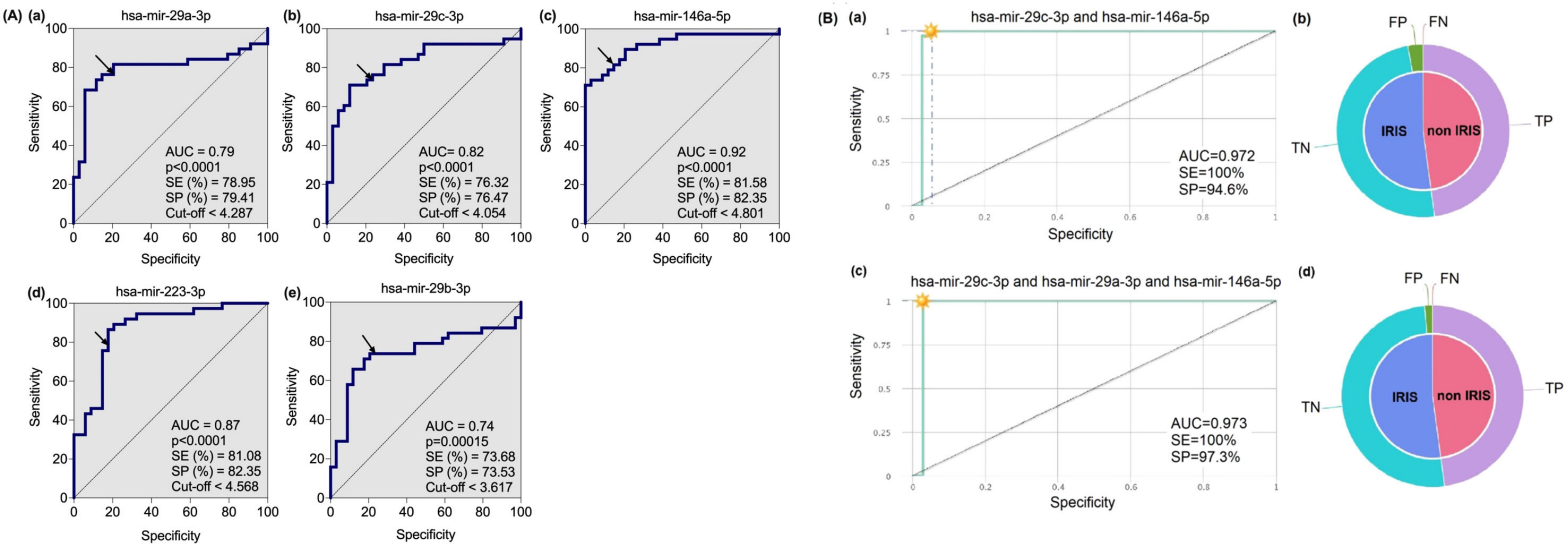
Receiver Operating characteristic (ROC) curve analysis of five microRNAs in IRIS versus non-IRIS patients at baseline. **(A)** Five plasma microRNAs that were highly significantly different between IRIS (n=35) and non-IRIS (n=39) were selected for ROC curve analysis using GraphPad Prism software version 10. The results of area under the curve (AUC), p-value, sensitivity (SE), specificity (SP) and cut-off (in log_10_MFI) for each microRNA marker are shown. The arrow on the curve indicates the point of a specific threshold with corresponding sensitivity and specificity. **(B)** The combinatorial ROC curve analysis of the five microRNAs was performed using the web-based application CombiROC (http://combiroc.eu/). Both combinatorial ROC curves have a sensitivity of 100% and a specificity of more than 94%. The proportions of false positive (FP), false negative (FN), true positive (TP) and true negative (TN) are shown in the pie chart.

For further in the analysis, we selected the five out of the six microRNAs from the volcano plot analysis (**Figure 2**, upper-left quadrant). The selection was made because these 5 miRNAs (hsa-mir-29a-3p; hsa-mir-29c-3p; hsa-mir-146a-5p; hsa-mir-223-3p; and hsa-mir-29b-3p) are highly significantly different (**Figure 4**). They also have less overlapping data of plasmatic miRNAs between IRIS and non-IRIS (see **Figure 1**). The individual receiver operating characteristic (ROC) curve analysis is shown in **Figure 4A**. All five microRNAs levels at baseline have a sensitivity and specificity greater than 74%. In addition, the area under the curve (AUC) values are greater than 0.74, indicating a higher accuracy of these individual microRNAs in predicting the onset of IRIS. To know whether a combination of selected microRNA markers could increase the sensitivity and specificity to predict IRIS, the combinatorial ROC curve analysis of the five selected microRNAs was performed using CombiROC (a free web-based application for guided and interactive generation of multi-marker panels). **Figure 4B** shows the best combinatorial analysis. The combination of two (hsa-mir-29c-3p and hsa-mir-146a-5p) (**Figure 4B**a, b) or three (hsa-mir-29c-3p; hsa-mir-29a-5p and hsa-mir-146a-5p) (**Figure 4B**c, d) has a sensitivity of 1.0 (no detection of false negatives), indicating that all IRIS cases can be predicted. In addition, very few false positives are detected, as suggested by the high specificity observed (0.946 and 0.973 for the two and three combinations, respectively).

## Discussion

IRIS is a state of inflammation that occurs during the first six months of antiretroviral treatment in HIV-infected people with ongoing opportunistic infections such as tuberculosis (15). Several risk factors for IRIS have been described (15). However, the presentation of IRIS can be heterogeneous and the diagnosis of IRIS in HIV co-infection is mainly clinical after exclusion of other diseases. Biological tests to aid in the clinical diagnosis of IRIS are still needed. Circulating miRNA are promising biomarkers for several diseases, including infectious diseases. In tuberculosis, microRNAs have been reported to modulate immune gene expression involving cellular-mediated immune response, inflammation; autophagy, and apoptosis (31, 32), and their differential expression, both circulating microRNAs and exosomal microRNAs, have been proposed as the potential biomarkers of tuberculosis (17, 18, 33, 34). In HIV infections, host microRNA can affect the HIV life cycle, and microRNAs profile was proposed as a predictive biomarker for HIV/AIDS disease progressions and monitoring of combined anti-retroviral drug treatment (35, 36). Olsson et al. have reported an alteration in the expression of a number of whole blood microRNAs in HIV-infected patients with active TB compared to TB without HIV infection (40). However, the study of the microRNA association with the onset of IRIS in individuals with HIV-TB co-infection is lacking.

We report here a distinct set of plasma microRNA expression levels at baseline, before anti-tuberculosis and combined anti-retroviral treatment, that can differentiate IRIS from non-IRIS in HIV-TB co-infected individuals. We found that 12 out of 26 microRNAs tested, known as anti-inflammatory microRNAs (37, 38) and involved in innate immune response (38), had significantly lower plasma levels in IRIS compared to non-IRIS individuals, suggesting a higher inflammatory response in the IRIS group. Notably, five of these 12 microRNAs (hsa-mir-29a-3p, hsa-mir-29c-3p; hsa-mir-146a-5p; hsa-mir-223-3p) have a highest scores as the best predictive marker in discriminating the two groups. Combinatory ROC analysis of these five microRNAs revealed that the association of two or three plasmatic miRNAs (hsa-mir-29c-3p, hsa-mir-29a-3p and hsa-mir-146-5p) highly predicts the occurrence of IRIS in HIV-TB co-infected individuals before any treatment, with 100% sensitivity and high specificity (95-97%). Thus, these molecular biomarkers can be used to identify and predict the onset of IRIS in HIV-TB co-infected individuals and could better guide the clinician in diagnosing the presence of an inflammatory syndrome after starting cART.

In contrast to plasmatic microRNAs, exosomal microRNA expression did not show a statistically significant difference between IRIS and non-IRIS individuals in our study. This may indicate that plasma microRNAs, but not exosomal microRNAs, play a prominent role in the occurrence of IRIS in HIV-TB co-infected individuals. Exosomal microRNAs have been the focus of several studies, as evidence suggests that the loading of microRNAs in exosomes is not a random process (26). Consequently, the measurement of exosomal microRNAs is considered a promising biomarker for various diseases, as the comparison of exosomal and plasma microRNA profiles in the same samples does not differ in healthy individuals (43). At the time of IRIS onset, although the expression levels of several plasma microRNAs, for instant hsa-mir-223-3p; hsa-mir-25-3p; hsa-mir-29a-3p; hsa-mir-29c-3p; and hsa-mir-145 were remaining decreased with statistically significantly different in IRIS compared to non-IRIS individuals, however, there was increased in fold change from baseline to the time of IRIS onset of several microRNAs in IRIS individuals, suggesting their role in controlling the inflammatory responses driven by tuberculosis associated IRIS.

It should be noted that the microRNAs in our study were selected to be mainly involved in innate immune responses and inflammation, as we and others have pointed out the role of innate immunity in the development of IRIS (39, 40). For instance, hsa-mir-146a-5p is a well-known regulator of innate immune activation pathway by inhibiting toll-like receptor and NF-κB signalling (41); while hsa-mir-223-3p, which was differentially expressed in our study, has been shown to negatively regulate excessive innate immune responses by controlling myeloid cells and macrophages activation; and NLRP3 inflammasome activity, thereby modulating IL-1β production (38), that is a reported cytokine significantly implicated in the aberrant inflammation associated with TB-IRIS (15). Also, miRNA-29 family members (hsa-mir-29a-3p; -29b-3p and -29c-3p) control immune responses by targeting interferon-gamma and other pro-inflammatory cytokines, suggesting its involvement interferon-gamma release during T-cell and NK-cell responses in the immunopathogenesis of TB-IRS (15, 42). The decreased of the level of miR-29 may lead to increase interferon gamma signalling, that is the hallmark of TB-IRIS. Taken together, IRIS individuals exhibit phenotypically associated with physiological inflammation mediated by anti-inflammatory microRNAs, whereas they were not observed in the case of non-IRIS participants, supporting the hypothesis that the modification of microRNA profiles may underlie unbalanced immune restoration and contribute to the development of TB-IRIS.

Our study has several limitations. Although the significant differences between IRIS and non-IRIS individuals in plasma levels of the 5 microRNAs are important, the size of our samples is not so large and therefore requires validation in a large cohort. Furthermore, the CAMELIA cohort was established in Cambodian population with HIV and TB with a high incidence of IRIS (27, 43). In addition, the values of plasmatic microRNA will need to be validated by other techniques such as PCR.

Although the key circulating microRNAs; for example hsa-mir-146a-5p; hsa-mir-29a-3p; hsa-mir-29c-3p; and hsa-mir-223-3p; were identified as predictive biomarkers of IRIS onset, their underlying mechanism in IRIS pathogenesis has yet to be explored.

In conclusion, we show here that a number of circulating microRNAs predicts the occurrence of IRIS in TB co-infected HIV individuals. These microRNAs are involved in the regulation of innate immunity and/or inflammation. Therefore, these results highlight and complement our previous studies on the role of innate immunity in the pathogenesis of IRIS.

There are still a lot of aspects that are in need of further investigation. Pulmonary tuberculosis induces an increase in a number of anti-inflammatory cytokines (44). The microRNA profile described here suggested that IRIS individuals appear to be able to control this anti-inflammatory response, whereas non-IRIS individuals cannot. Furthermore, after TB treatment and as reported for cytokines (44), non-IRIS individuals decreased plasmatic anti-inflammatory microARNs. In the case of IRIS, they increased plasmatic levels of anti-inflammatory microARN, perhaps to try to control their inflammatory response after clinical manifestations. The delicate balance between inflammation and non-inflammation needs to be further explored in the context of IRIS management.

## Author’s note

The findings described in this study have been accepted for a patent NoNT/NG/IDM-22-0055, filed by Institut Pasteur/Scott-Algara et al.

## Data Availability

The original contributions presented in the study are included in the article/**Supplementary Material**. Further inquiries can be directed to the corresponding author.
