# Supplementary material for: A set of plasmatic microRNA related to innate immune response highly predicts the onset of immune reconstitution inflammatory syndrome in tuberculosis co-infected HIV individuals (ANRS-12358 study)
Source: Front Immunol. 2025 Jun 20;16:1603338. doi: 10.3389/fimmu.2025.1603338 (PMC12231349; doi:10.3389/fimmu.2025.1603338)
Supplement: Supplementary file 1 [file DataSheet1.docx]

**Supplementary figures**


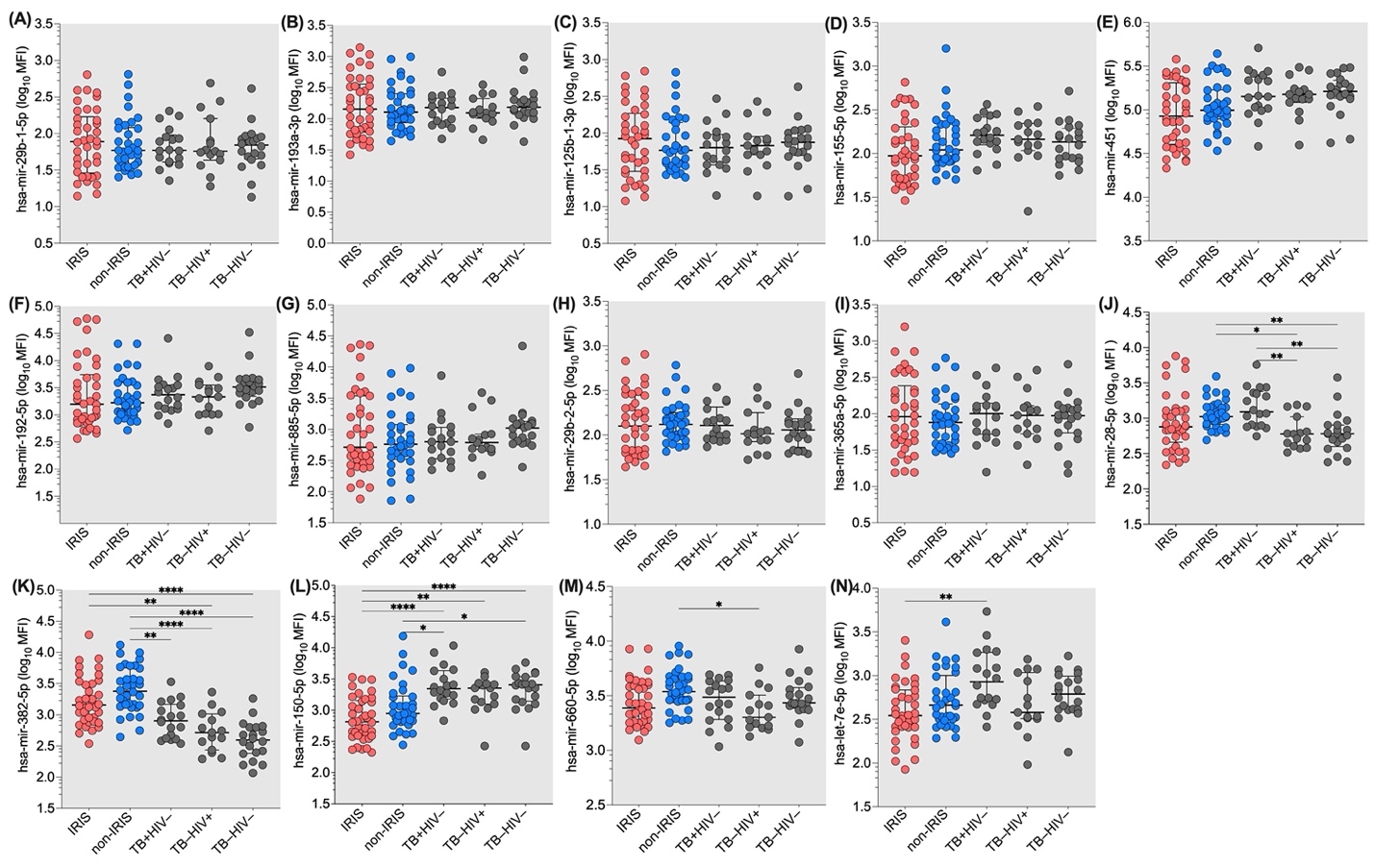


**Figure S1. Comparison of the expression levels of fourteen plasma microRNAs in IRIS and non-IRIS patients at baseline and controls.** The expression levels of the fourteen plasma microRNAs were not significantly different between IRIS and non-IRIS at baseline. Nine plasma microRNAs were similar between IRIS and non-IRIS and control groups. The Y-axis represents the level of plasma microRNA in logarithmic mean fluorescence intensity (log_10_ MFI). The X-axis represents the groups studied. Kruskal-Wallis test was used for statistical analysis. Results are presented as median (25%-75% IQR) log10MFI. The statistically significant difference in the comparison between different study groups is indicated: * p<0.05; ** p=0.01; *** p=0.001; and **** p<;0.0001.


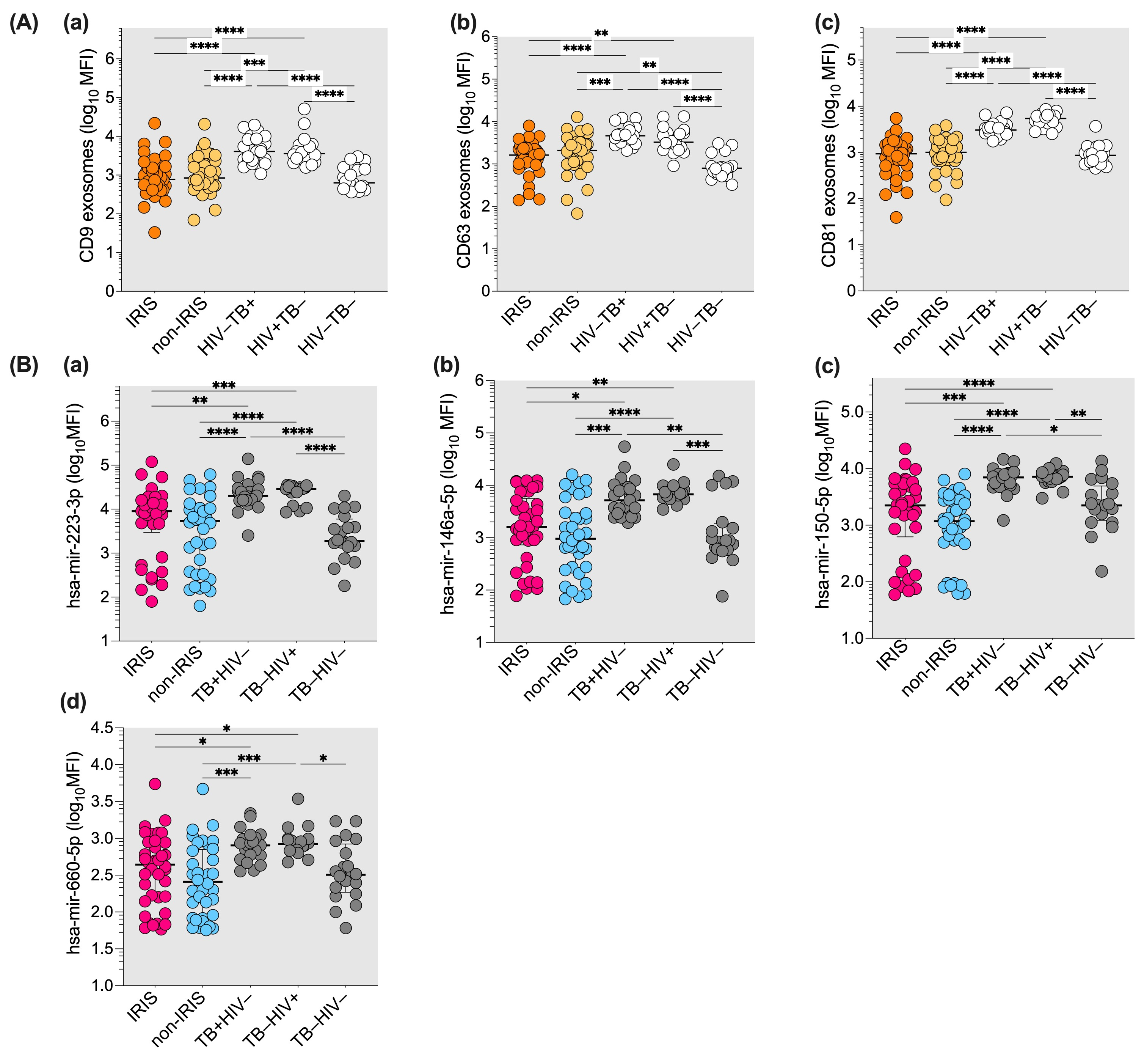


**Figure S2. Plasma exosomes and exosomal microRNAs content in IRIS and non-IRIS patients at baseline and controls.** Exosomes were isolated and purified by size exclusion chromatography and filter centrifugation, respectively. (A) Exosomal markers CD9 (a), CD63 (b) and CD81 (c) were labelled with fluorochrome-conjugated monoclonal antibodies and analysed by flow cytometry. (B) Four exosomal microRNA levels in the studied groups are shown. The Y-axis represents the level of exomiR in logarithmic mean fluorescence intensity (log10 MFI). The X-axis represents the study groups. Kruskal-Wallis test was used for analysis. Results are presented as median (25%-75% IQR) log10MFI. Statistically significant differences between groups are indicated: * p<0.05; ** p=0.01; *** p=0.001; and **** p<0.0001.

**
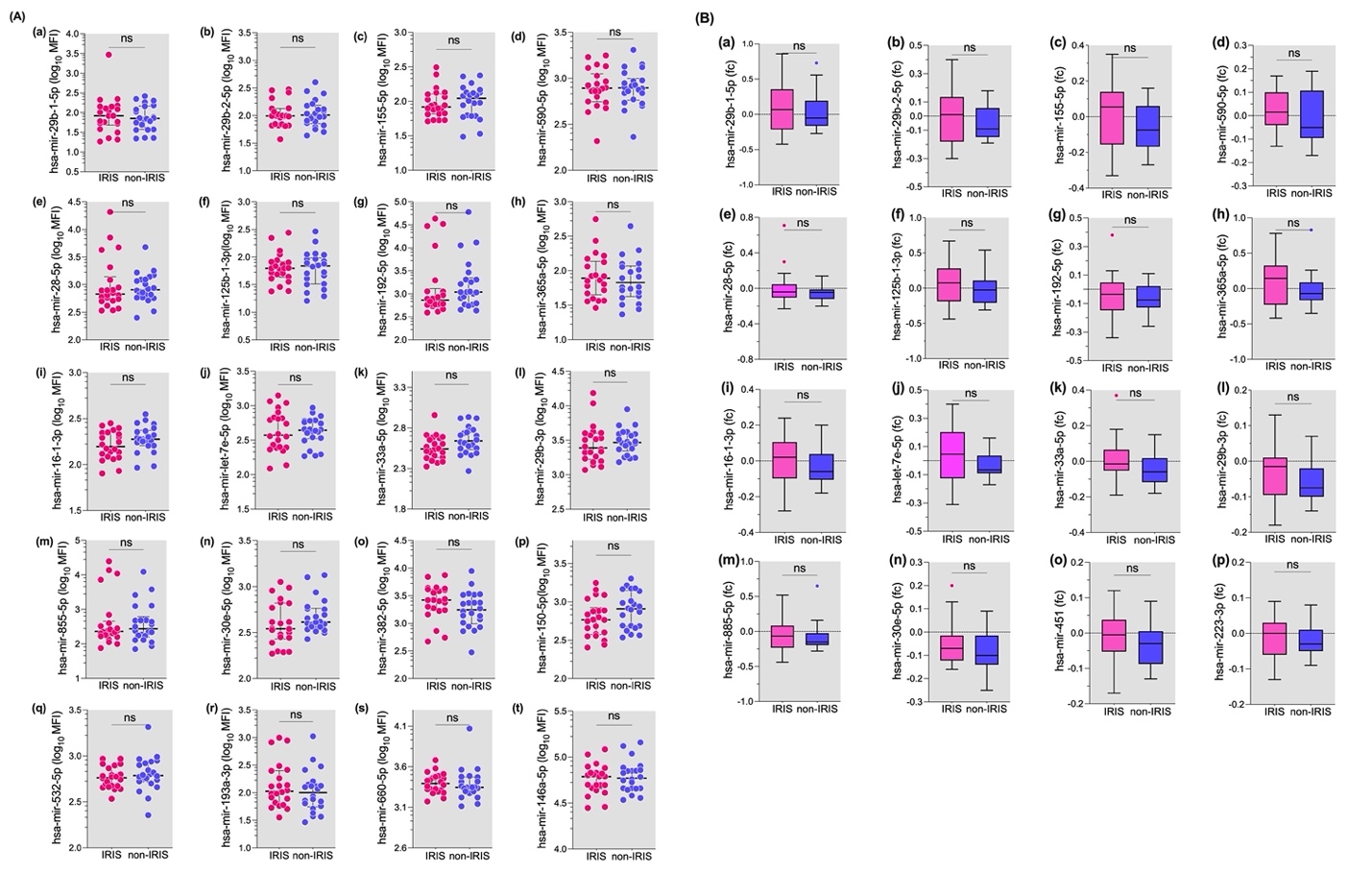
**

**Figure S3. Levels of plasmatic microRNAs and their fold changes at IRIS onset.**

(A) At IRIS onset, 20 out of 26 microRNAs tested showed no statistically significant differences between IRIS and non-IRIS patients. (B) While the fold changes of 16 microRNAs from baseline to IRIS onset were not statistically significantly different between IRIS and non-IRIS patients, eight microRNAs showed a negative fold change in both groups. Conversely, seven microRNAs showed positive fold changes in IRIS patients but negative changes in non-IRIS controls. The Wilcoxon matched-pair test was used for statistical analysis and results are presented as medians with interquartile ranges (25%-75% IQR). "ns" indicates no significant difference.


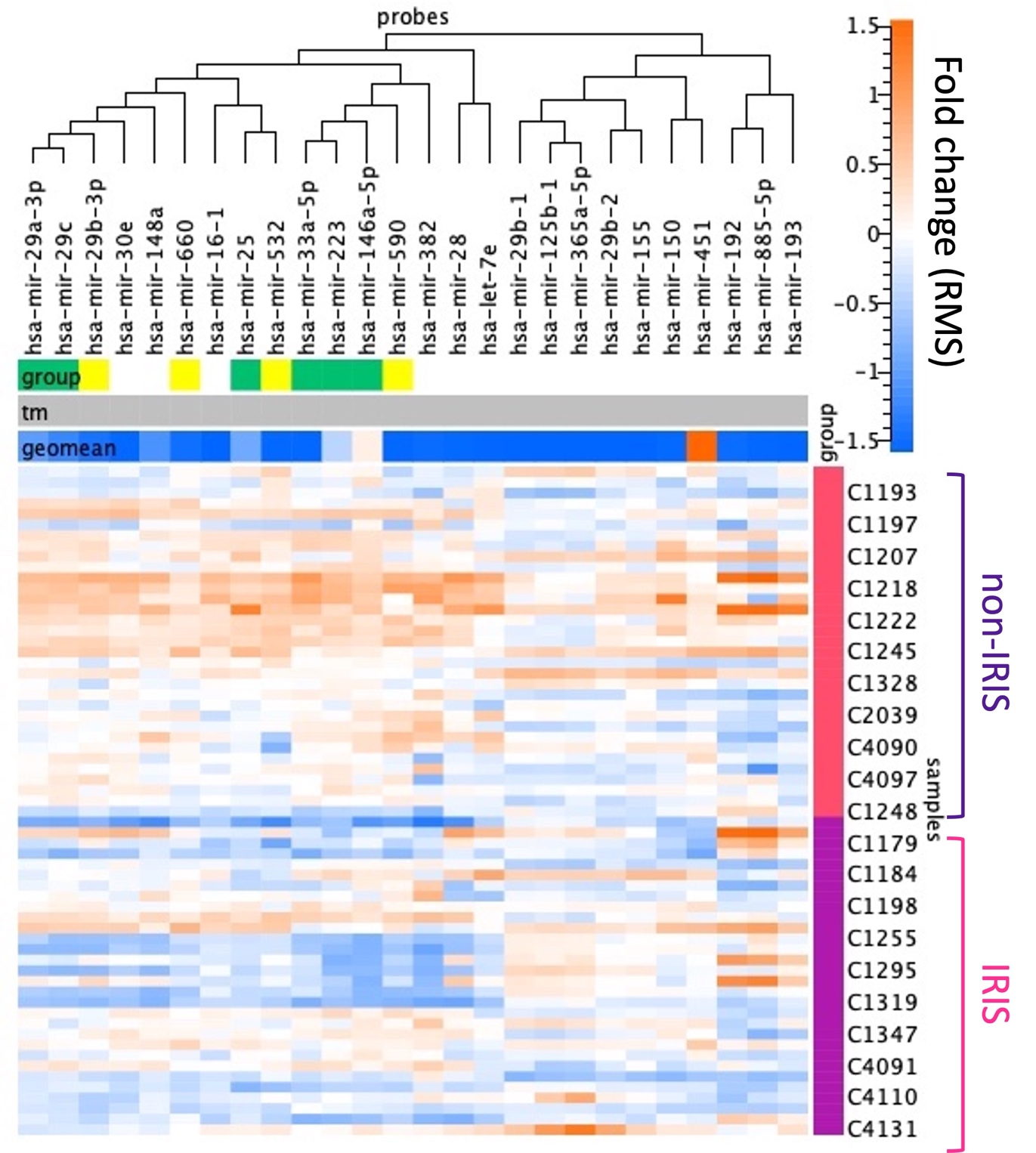


**Figure S4. Heatmap comparing the expression levels of 26 tested plasmatic microRNAs in IRIS and non-IRIS patients at baseline.** The probe at the top represents the names of the 26 tested microRNAs analysed in the heatmap and hierarchically clustered based on their plasma levels patterns. The green (p-value=0.0002) and yellow (p-value=0.02) coloured horizontal bar shows the group of microRNAs whose plasma levels are significantly different between IRIS and non-IRIS patients at baseline. The vertical bar on the right shows the ID of the patients: non-IRIS group (red) and IRIS group (purple). Each cell in the heatmap represents the fold change of plasma level for each microRNA. The upper bar on the right indicate high and low plasma level of each microRNAs, according to gradiant of orange or blue colours. The heatmap was generated using Fireplex Analysis Work Bench software (Abcam, London, UK).
