# Supplementary material for: A set of plasmatic microRNA related to innate immune response highly predicts the onset of immune reconstitution inflammatory syndrome in tuberculosis co-infected HIV individuals (ANRS-12358 study)
Source: Front Immunol. 2025 Jun 20;16:1603338. doi: 10.3389/fimmu.2025.1603338 (PMC12231349; doi:10.3389/fimmu.2025.1603338)
Supplement: Supplementary file 2 [file Table1.docx]

| **Previous ID** | **Mature Name** | **Accession #** | **Mature sequence** | **Chr** | **Target** | **Function** | | **References** | |
| --- | --- | --- | --- | --- | --- | --- | --- | --- | --- |
| hsa-let-7e-5p | hsa-let-7e-5p | MIMAT0000066 | UGAGGUAGGAGGUUGUAUAGUU | Chr19 | *TLR4*  *CD14*; *TNFA;* *IL-6* | Negative regulator of  NF-κB pathway | | (Kumar et al. 2015, Ren and Ambros 2015, Curtale et al. 2018) | |
| hsa-mir-16-1* | hsa-mir-16-1-3p | MIMAT0004489 | CCAGUAUUAACUGUGCUGCUGA | Chr13 | NF-κB1; IL6; CXCL8; TNF; TAB2 | Inhibition of NF-κB pathway. Suppression of pro-inflammatory cytokine responses. Regulate leukocyte recruitment | | miRTarBase (prediction) | |
| hsa-miR-25-3p | hsa-miR-25-3p | MIMAT0000081 | CAUUGCACUUGUCUCGGUCUGA | Chr7 | *TLR4* | Inhibition NLRP3/IL-1β/IL-8 pathway | | (Luo et al. 2022) | |
| hsa-miR-28-5p | hsa-miR-28-5p | MIMAT0000085 | AAGGAGCUCACAGUCUAUUGAG | Chr3 | IRAK4; *N4BP1; IRF* | Inhibition of NF-κB pathway. Modulation of macrophage polarization  Regulation of TLR and type I IFN pathway | | Target Scan (Prediction) | |
| hsa-miR-29a | hsa-miR-29a-3p | MIMAT0000086 | UAGCACCAUCUGAAAUCGGUUA | Chr7 | *T-BET*, *EOMES*, *IFNG; NFIA; GPR85; SOCS-1; PI3K* | Promote M2 macrophage polarization  Modulation of Interferon signaling pathway  Suppression of IFNγ production | | (Yao et al. 2025) | |
| hsa-miR-29b-1 | hsa-miR-29b-1-5p | MIMAT0004514 | GCUGGUUUCAUAUGGUGGUUUAGA | Chr7 | *PTX3; DNMT3A; DNMT3B; STAT3;  NF-κB* | Regulation of inflammatory response (anti-inflammatory effect) | | (Ma et al. 2011, Botta et al. 2018, Abdalla et al. 2023) | |
| hsa-miR-29b-2 | hsa-miR-29b-2-5p | MIMAT0004515 | CUGGUUUCACAUGGUGGCUUA | Chr1 | *C1QTNF6* | Inhibition of inflammatory responses by suppression CRP, IL-IL-6 and TNFα | | (Botta et al. 2018, Ma et al. 2022, Abdalla et al. 2023) | |
| hsa-miR-29c | hsa-miR-29c-3p | MIMAT0000681 | UAGCACCAUUUGAAAUCGGUUA | Chr1 | *TNFAIP3; TGFBR2; STAT3; SP1* | Negative regulation of NF-κB and cytokines signaling. Modulation of macrophage activation. | | (Abdalla et al. 2023) | |
| hsa-miR-29b-3p | hsa-miR-29b-3p | MIMAT0000100 | UAGCACCAUUUGAAAUCAGUGUU | Chr7 | *TGFBR1 / TGFBR2; STAT3; SP1; TLR2; IL23A* | Modulation of cytokine production. Inhibition of PRR signaling | | (Ma et al. 2011) | |
| hsa-miR-30e | hsa-miR-30e-5p | MIMAT0000692 | UGUAAACAUCCUUGACUGGAAG | Chr1 | *SOCS; TRIM38;* | Negative regulator of RIG-1 signaling  Inhibition JAK/STAT signaling.  Positive regulator of innate immune signaling | | (Lin et al. 2020, Mishra et al. 2020) | |
| hsa-miR-33a-5p | hsa-miR-33a-5p | MIMAT0000091 | GUGCAUUGUAGUUGCAUUGCA | Chr22 | *IRAK1; STING;*  *ABCA1, NLRP3* | TLR/IL-1R signaling  Pro-inflammatory effect | (Nejad et al. 2018) | |  |
| hsa-miR-125b-1 | hsa-miR-125b-1-3p | MIMAT0004592 | ACGGGUUAGGCUCUUGGGAGCU | Chr11 | *TNFA; TRAF6; A20; IRF4* | Modulation of macrophage activation  Negative co-regulator of inflammatory genes | (Tili et al. 2007, Chaudhuri et al. 2011, Valmiki et al. 2019) | |  |
| hsa-miR-146a-5p | hsa-miR-146a-5p | MIMAT0000449 | UGAGAACUGAAUUCCAUGGGUU | Chr5 | *TRAF6,*  *IRAK1* | Negative regulator of inflammation | (Taganov et al. 2006, Saba et al. 2014) | |  |
| hsa-miR-148a | hsa-miR-148a-3p | MIMAT0000243 | UCAGUGCACUACAGAACUUUGU | Chr7 | *Notch*  *PTEN* | Promote M1 macrophage polarization | (Huang et al. 2017) | |  |
| hsa-miR-150 | hsa-miR-150-5p | MIMAT0000451 | UCUCCCAACCCUUGUACCAGUG | Chr19 | *STAT1* | Anti-inflammatory effect | (Chen et al. 2021) | |  |
| hsa-miR-155 | hsa-miR-155-5p | MIMAT0000646 | UUAAUGCUAAUCGUGAUAGGGGUU | Chr21 | *TAB2, MyD88, IKKε, Ripk1, C/EBPβ, eNOS, p65, SHIP1, SOCS1* | Regulation of inflammatory cytokines production | (Ceppi et al. 2009, Li et al. 2022, Abdalla et al. 2023) | |  |
| hsa-miR-192 | hsa-miR-192-5p | MIMAT0000222 | CUGACCUAUGAAUUGACAGCC | Chr11 | *CXCL2; IL-1RI;* | Regulation of chemokine production and cytokine signaling | (Raisch et al. 2013, Ren et al. 2021) | |  |
| hsa-miR-193 | hsa-miR-193a-3p | MIMAT0000459 | AACUGGCCUACAAAGUCCCAGU | Chr17 | *TNFRSF21; IL6ST; VNN1; FADS1* | Modulation of NF-κB pathway; Regulation of IL-6 signaling; Macrophage polarization | (Chen et al. 2017, Gong et al. 2023) | |  |
| hsa-miR-223 | hsa-miR-223-3p | MIMAT0000280 | UGUCAGUUUGUCAAAUACCCCA | ChrX | *NLRP3; STAT3; RhoB; IRAK1; FOXO3; IKKα* | Inhibition of inflammasome activation; modulation of cytokines production; Attenuation of TLR/IL-1R signaling | (Yuan et al. 2018, Yuan et al. 2021) | |  |
| hsa-miR-365a-5p | hsa-miR-365a-5p | MIMAT0009199 | AGGGACUUUUGGGGGCAGAUGUG | Chr16 | *IL-6; BCL2; BAX; ARRB2* | Regulation of cytokines production; modulation of apoptosis; T cell migration ; chemotactic responses | (Davuluri and Chauhan 2022, Wang et al. 2022) | |  |
| hsa-miR-382 | hsa-miR-382-5p | MIMAT0000737 | GAAGUUGUUCGUGGUGGAUUCG | Chr14 | *CDK8; CXCL12; PGC-1α* | Modulation of macrophage polarization and immune cell recruitment | (Lv et al. 2021) | |  |
| hsa-miR-451 | hsa-miR-451a | MIMAT0001631 | AAACCGUUACCAUUACUGAGUU | Chr17 | *MIF; CAB39; CXCL16; ATF2* | Suppress of pro-inflammatory cytokines production; regulation of macrophages polarization and migration | (Bandres et al. 2009, Rosenberger et al. 2012) | |  |
| hsa-miR-532 | hsa-miR-532-5p | MIMAT0002888 | CAUGCCUUGAGUGUAGGACCGU | ChrX | *IRAK1, TRAF6* | Regulation of NF-κB pathway | (Li et al. 2022) | |  |
| hsa-miR-590 | hsa-miR-590-5p | MIMAT0003258 | GAGCUUAUUCAUAAAAGUGCAG | Chr7 | *SMAD3* | Negative regulation of TGFβ signaling pathway | (Jafarzadeh and Soltani 2016) | |  |
| hsa-miR-660 | hsa-miR-660-5p | MIMAT0003338 | UACCCAUUGCAUAUCGGAGUUG | ChrX | *MDM2*  *and p53* | Indirectly negative regulation of NFκB pathway | (Lin et al. 2023, Wang et al. 2025) | |  |
| hsa-miR-885-5p | hsa-miR-885-5p | MIMAT0004947 | UCCAUUACACUACCCUGCCUCU | Chr-3 | *SOCS* | Regulation of NF-κB pathway  Modulation of TLR signaling  Inhibition of cytokine signaling | (Su et al. 2018, Zhang et al. 2020, Sun et al. 2022) | |  |

**Table S1. List of human microRNAs used in the experiments**

The twenty-six microRNAs were selected by searching MEDLINE, Scopus and Web of Science libraries for scientific journal publications between 1 January 2010 and 31 December 2016. The microRNA sequence, accession number and chromosomal loci were obtained from the miRbase online resource for human microRNA sequence and annotation (v 22.1) (http://mirbase.org/).

**References**

Abdalla, A. E., A. Alanazi, K. O. A. Abosalif, A. A. M. Alameen, K. Junaid, E. Manni, A. A. Talha and H. Ejaz (2023). "MicroRNA-155, a double-blade sword regulator of innate tuberculosis immunity." Microb Pathog **185**: 106438.

Bandres, E., N. Bitarte, F. Arias, J. Agorreta, P. Fortes, X. Agirre, R. Zarate, J. A. Diaz-Gonzalez, N. Ramirez, J. J. Sola, P. Jimenez, J. Rodriguez and J. Garcia-Foncillas (2009). "microRNA-451 regulates macrophage migration inhibitory factor production and proliferation of gastrointestinal cancer cells." Clin Cancer Res **15**(7): 2281-2290.

Botta, C., M. Cuce, M. R. Pitari, D. Caracciolo, A. Gulla, E. Morelli, C. Riillo, L. Biamonte, M. E. Gallo Cantafio, R. Prabhala, C. Mignogna, A. Di Vito, E. Altomare, N. Amodio, M. T. Di Martino, P. Correale, M. Rossi, A. Giordano, N. C. Munshi, P. Tagliaferri and P. Tassone (2018). "MiR-29b antagonizes the pro-inflammatory tumor-promoting activity of multiple myeloma-educated dendritic cells." Leukemia **32**(4): 1003-1015.

Ceppi, M., P. M. Pereira, I. Dunand-Sauthier, E. Barras, W. Reith, M. A. Santos and P. Pierre (2009). "MicroRNA-155 modulates the interleukin-1 signaling pathway in activated human monocyte-derived dendritic cells." Proc Natl Acad Sci U S A **106**(8): 2735-2740.

Chaudhuri, A. A., A. Y. So, N. Sinha, W. S. Gibson, K. D. Taganov, R. M. O'Connell and D. Baltimore (2011). "MicroRNA-125b potentiates macrophage activation." J Immunol **187**(10): 5062-5068.

Chen, Q., C. Tong, S. Ma, L. Zhou, L. Zhao and X. Zhao (2017). "Involvement of MicroRNAs in Probiotics-Induced Reduction of the Cecal Inflammation by Salmonella Typhimurium." Front Immunol **8**: 704.

Chen, S., H. Zhu, J. Sun, L. Zhu, L. Qin and J. Wan (2021). "Anti-inflammatory effects of miR-150 are associated with the downregulation of STAT1 in macrophages following lipopolysaccharide treatment." Exp Ther Med **22**(4): 1049.

Curtale, G., T. A. Renzi, M. Mirolo, L. Drufuca, M. Albanese, M. De Luca, M. Rossato, F. Bazzoni and M. Locati (2018). "Multi-Step Regulation of the TLR4 Pathway by the miR-125a~99b~let-7e Cluster." Front Immunol **9**: 2037.

Davuluri, K. S. and D. S. Chauhan (2022). "microRNAs associated with the pathogenesis and their role in regulating various signaling pathways during Mycobacterium tuberculosis infection." Front Cell Infect Microbiol **12**: 1009901.

Gong, Q., H. Li, J. Song and C. Lin (2023). "LncRNA LINC01569 promotes M2 macrophage polarization to accelerate hypopharyngeal carcinoma progression through the miR-193a-5p/FADS1 signaling axis." J Cancer **14**(9): 1673-1688.

Huang, F., J. L. Zhao, L. Wang, C. C. Gao, S. Q. Liang, D. J. An, J. Bai, Y. Chen, H. Han and H. Y. Qin (2017). "miR-148a-3p Mediates Notch Signaling to Promote the Differentiation and M1 Activation of Macrophages." Front Immunol **8**: 1327.

Jafarzadeh, M. and B. M. Soltani (2016). "Hsa-miR-590-5p Interaction with SMAD3 Transcript Supports Its Regulatory Effect on The TGFbeta Signaling Pathway." Cell J **18**(1): 7-12.

Kumar, M., S. K. Sahu, R. Kumar, A. Subuddhi, R. K. Maji, K. Jana, P. Gupta, J. Raffetseder, M. Lerm, Z. Ghosh, G. van Loo, R. Beyaert, U. D. Gupta, M. Kundu and J. Basu (2015). "MicroRNA let-7 modulates the immune response to Mycobacterium tuberculosis infection via control of A20, an inhibitor of the NF-kappaB pathway." Cell Host Microbe **17**(3): 345-356.

Li, C., L. Huang, Y. Wen, M. Yi and M. Gao (2022). "Bioinformatics Analysis of miRNAs Targeting TRAF5 in DLBCL Involving in NF-kappaB Signaling Pathway and Affecting the Apoptosis and Signal Transduction." Genet Res (Camb) **2022**: 3222253.

Lin, M., M. Liu, X. Han, X. Tao, Z. Tang and Q. Ma (2023). "The role of differentially expressed miR-660 in peripheral blood lymphocytes of patients with pulmonary tuberculosis." Biomarkers **28**(5): 409-415.

Lin, X., S. Yu, P. Ren, X. Sun and M. Jin (2020). "Human microRNA-30 inhibits influenza virus infection by suppressing the expression of SOCS1, SOCS3, and NEDD4." Cell Microbiol **22**(5): e13150.

Luo, X. Y., J. H. Ying and Q. S. Wang (2022). "miR-25-3p ameliorates SAE by targeting the TLR4/NLRP3 axis." Metab Brain Dis **37**(6): 1803-1813.

Lv, Y., Y. Li, J. Wang, M. Li, W. Zhang, H. Zhang, Y. Shen, C. Li, Y. Du and L. Jiang (2021). "MiR-382-5p suppresses M1 macrophage polarization and inflammatory response in response to bronchopulmonary dysplasia through targeting CDK8: Involving inhibition of STAT1 pathway." Genes Cells **26**(10): 772-781.

Ma, F., S. Xu, X. Liu, Q. Zhang, X. Xu, M. Liu, M. Hua, N. Li, H. Yao and X. Cao (2011). "The microRNA miR-29 controls innate and adaptive immune responses to intracellular bacterial infection by targeting interferon-gamma." Nat Immunol **12**(9): 861-869.

Ma, X., H. J. Yun, K. Elkin, Y. Guo, Y. Ding and G. Li (2022). "MicroRNA-29b Suppresses Inflammation and Protects Blood-Brain Barrier Integrity in Ischemic Stroke." Mediators Inflamm **2022**: 1755416.

Mishra, R., S. Bhattacharya, B. S. Rawat, A. Kumar, A. Kumar, K. Niraj, A. Chande, P. Gandhi, D. Khetan, A. Aggarwal, S. Sato, P. Tailor, A. Takaoka and H. Kumar (2020). "MicroRNA-30e-5p has an Integrated Role in the Regulation of the Innate Immune Response during Virus Infection and Systemic Lupus Erythematosus." iScience **23**(7): 101322.

Nejad, C., H. J. Stunden and M. P. Gantier (2018). "A guide to miRNAs in inflammation and innate immune responses." FEBS J **285**(20): 3695-3716.

Raisch, J., A. Darfeuille-Michaud and H. T. Nguyen (2013). "Role of microRNAs in the immune system, inflammation and cancer." World J Gastroenterol **19**(20): 2985-2996.

Ren, F. J., Y. Yao, X. Y. Cai and G. Y. Fang (2021). "Emerging Role of MiR-192-5p in Human Diseases." Front Pharmacol **12**: 614068.

Ren, Z. and V. R. Ambros (2015). "Caenorhabditis elegans microRNAs of the let-7 family act in innate immune response circuits and confer robust developmental timing against pathogen stress." Proc Natl Acad Sci U S A **112**(18): E2366-2375.

Rosenberger, C. M., R. L. Podyminogin, G. Navarro, G. W. Zhao, P. S. Askovich, M. J. Weiss and A. Aderem (2012). "miR-451 regulates dendritic cell cytokine responses to influenza infection." J Immunol **189**(12): 5965-5975.

Saba, R., D. L. Sorensen and S. A. Booth (2014). "MicroRNA-146a: A Dominant, Negative Regulator of the Innate Immune Response." Front Immunol **5**: 578.

Su, M., B. Qin, F. Liu, Y. Chen and R. Zhang (2018). "miR-885-5p upregulation promotes colorectal cancer cell proliferation and migration by targeting suppressor of cytokine signaling." Oncol Lett **16**(1): 65-72.

Sun, J., J. Wei, Y. Zhang, J. Li, J. Li, J. Yan, M. Guo, J. Han and H. Qiao (2022). "Plasma Exosomes Transfer miR-885-3p Targeting the AKT/NFkappaB Signaling Pathway to Improve the Sensitivity of Intravenous Glucocorticoid Therapy Against Graves Ophthalmopathy." Front Immunol **13**: 819680.

Taganov, K. D., M. P. Boldin, K. J. Chang and D. Baltimore (2006). "NF-kappaB-dependent induction of microRNA miR-146, an inhibitor targeted to signaling proteins of innate immune responses." Proc Natl Acad Sci U S A **103**(33): 12481-12486.

Tili, E., J. J. Michaille, A. Cimino, S. Costinean, C. D. Dumitru, B. Adair, M. Fabbri, H. Alder, C. G. Liu, G. A. Calin and C. M. Croce (2007). "Modulation of miR-155 and miR-125b levels following lipopolysaccharide/TNF-alpha stimulation and their possible roles in regulating the response to endotoxin shock." J Immunol **179**(8): 5082-5089.

Valmiki, S., V. Ahuja, N. Puri and J. Paul (2019). "miR-125b and miR-223 Contribute to Inflammation by Targeting the Key Molecules of NFkappaB Pathway." Front Med (Lausanne) **6**: 313.

Wang, W., Y. Li, J. Fan, X. Qu, D. Shang, Q. Qin, T. Xu, Q. Hamid, X. Dang, Y. Chang and D. Xu (2022). "MiR-365-3p is a negative regulator in IL-17-mediated asthmatic inflammation." Front Immunol **13**: 953714.

Wang, Z., X. Su, Z. Zhan, H. Wang, S. Zhou, J. Mao, H. Xu and S. Duan (2025). "miR-660: A novel regulator in human cancer pathogenesis and therapeutic implications." Gene **953**: 149434.

Yao, X. C., J. J. Wu, S. T. Yuan and F. L. Yuan (2025). "Recent insights and perspectives into the role of the miRNA‑29 family in innate immunity (Review)." Int J Mol Med **55**(3).

Yuan, S., Q. Wu, Z. Wang, Y. Che, S. Zheng, Y. Chen, X. Zhong and F. Shi (2021). "miR-223: An Immune Regulator in Infectious Disorders." Front Immunol **12**: 781815.

Yuan, X., N. Berg, J. W. Lee, T. T. Le, V. Neudecker, N. Jing and H. Eltzschig (2018). "MicroRNA miR-223 as regulator of innate immunity." J Leukoc Biol **104**(3): 515-524.

Zhang, X., H. Gu, L. Wang, F. Huang and J. Cai (2020). "MiR-885-3p is down-regulated in peripheral blood mononuclear cells from T1D patients and regulates the inflammatory response via targeting TLR4/NF-kappaB signaling." J Gene Med **22**(1): e3145.
